# Unbiased Prediction and Feature Selection in High-Dimensional Survival Regression

**DOI:** 10.1089/cmb.2015.0192

**Published:** 2016-04-01

**Authors:** Michael Laimighofer, Jan Krumsiek, Florian Buettner, Fabian J. Theis

**Affiliations:** ^1^ Institute of Computational Biology, Helmholtz-Zentrum München, Neuherberg, Germany.; ^2^ Department of Mathematics, TU München, Garching, Germany.; ^3^ German Center for Diabetes Research (DZD), München-Neuherberg, Germany.; ^4^ European Bioinformatics Institute, European Molecular Biology Laboratory Hinxton, Cambridge, United Kingdom.

**Keywords:** feature selection, high-dimensional survival regression, repeated nested cross validation

## Abstract

**With widespread availability of omics profiling techniques, the analysis and interpretation of high-dimensional omics data, for example, for biomarkers, is becoming an increasingly important part of clinical medicine because such datasets constitute a promising resource for predicting survival outcomes. However, early experience has shown that biomarkers often generalize poorly. Thus, it is crucial that models are not overfitted and give accurate results with new data. In addition, reliable detection of multivariate biomarkers with high predictive power (feature selection) is of particular interest in clinical settings. We present an approach that addresses both aspects in high-dimensional survival models. Within a nested cross-validation (CV), we fit a survival model, evaluate a dataset in an unbiased fashion, and select features with the best predictive power by applying a weighted combination of CV runs. We evaluate our approach using simulated toy data, as well as three breast cancer datasets, to predict the survival of breast cancer patients after treatment. In all datasets, we achieve more reliable estimation of predictive power for unseen cases and better predictive performance compared to the standard CoxLasso model. Taken together, we present a comprehensive and flexible framework for survival models, including performance estimation, final feature selection, and final model construction. The proposed algorithm is implemented in an open source R package (SurvRank) available on CRAN.**

## 1. Introduction

In past years, new experimental technologies that allow measurement of tens of thousands of SNPs, transcripts, peptides, and metabolites in a cost-effective, high-throughput fashion have been developed. Consequently, omics measurements in patient samples are increasingly becoming part of clinical trials (McShane et al., 2013), because they promise to serve diagnostic purposes and accurately model patient survival times. However, for such survival models to be adopted in clinical practice and diagnosis, it is crucial to accurately estimate the generalizability of these models (i.e., how well they perform with new patient cohorts). In addition, identification of a small set of highly predictive features in a high-dimensional survival setting is of particular clinical interest as it can facilitate large-scale screening of large patient cohorts. Example applications include identification of genetic marker sets to predict survival times after surgery in cancer research (Desmedt et al., 2007; van de Vijver et al., 2002) and the prediction of time to diabetes onset (Abbasi et al., 2012).

In high-dimensional medical datasets, the number of features *p* usually far exceeds the number of observations *n* (*n << p*). Several previous studies have addressed the *n << p* problem in survival settings using regularization or feature selection approaches. Some authors have combined test statistics from univariate analyses into risk scores, for example, for lung cancer (Beer et al., 2002) and colorectal cancer (Eschrich et al., 2005). A drawback of these approaches is that each feature is individually associated with survival; however, joint information across features is not used. With polygenic risk scores or multivariate biomarkers, interest in full multivariable models has increased. As standard regression-based models are prone to overfitting in the *n << p* scenario, shrinkage-based models, which regularize the effect estimates, are commonly used (Gui and Li, 2005; Wu et al., 2011; Gong et al., 2014; Datta et al., 2007). Alternatively, dimensionality reduction (e.g., PCA or clustering) can be performed prior to survival modeling (Alizadeh et al., 2000; Takamizawa et al., 2004; Zhao et al., 2005).

Here, we propose an approach that tackles two major challenges for predictive survival models in a single unified algorithm. TASK 1: A predictor must show good generalizability, that is it must correctly predict an outcome using unseen observations. Here, we aim to obtain unbiased predictions using only training data, that is in the absence of a validation dataset. The generalizability of this type of prediction model can be quantified using measures such as the concordance index (C-index) within a cross-validation (CV) framework for survival data (Harrell et al., 1982). For applicability in clinical settings, it is crucial to estimate this predictive power for new, unseen patients in an unbiased fashion. TASK 2: We aim to select a reduced set of informative features that retains high predictive accuracy. While different approaches to address these tasks in binary classification settings exist, to the best of our knowledge, there is no unified framework for high-dimensional survival settings.

**FIG. 1.**
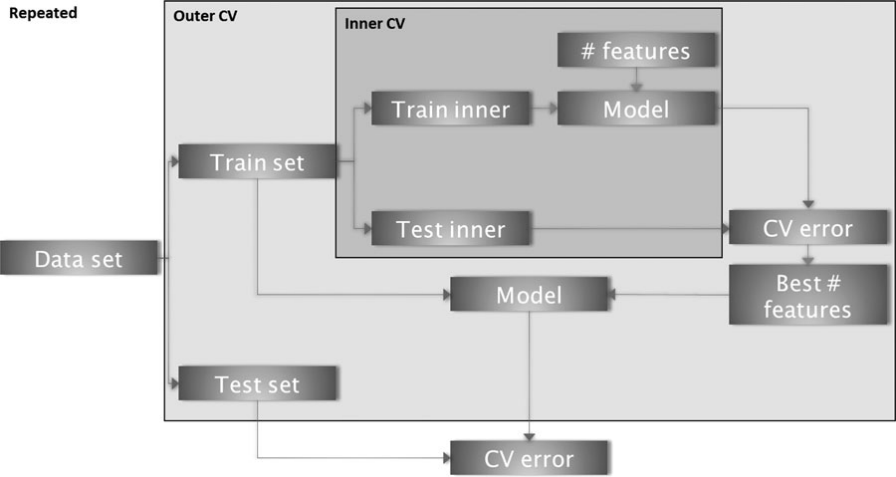
Overview of the repeated nested Cross-validation scheme. In the inner CV, the optimal number of parameters is determined. The outer CV loop estimates unbiased prediction accuracy. The variance of the prediction accuracy is estimated by repeating the entire procedure.

We use a repeated nested CV strategy to tackle both tasks (Fig. 1). Specifically, we use a feature ranking-based approach to perform model selection followed by determination of the optimal number of features in the inner CV loop. The outer CV is used to estimate the prediction accuracy with the C-index with unseen data. By repeating the entire procedure, we quantify the intrinsic variation in the prediction accuracy. As different CV folds will produce different lists of feature rankings, we propose an algorithm to combine results. We weight the features according to their performance in the CV. TASK 1 is addressed by our method due to the strict separation of the training and test sets. We solve TASK 2 using our proposed approach to aggregate CV information into a final set of features.

We evaluate our approach with simulated data with a fixed set of features and show that existing methods (a regularized survival Cox model) exhibit strong bias. In addition, we test performance with three publicly available breast cancer datasets. These microarray-based datasets contain gene expression data from patients with lymph node-negative breast cancer after surgery or radiotherapy. Our pipeline is available as an R package (R Core, Team, 2014) SurvRank online.

## 2. Methods

A survival dataset is defined by the triple (*T_i_*, *δ_i_*, **x***_i_*) *i* = 1*,…,n* subjects, where *T_i_* is the observed time (either failure time or censoring time), *δ_i_*∈{0, 1} denotes the censoring indicator for a failure event (e.g., *δ_i_* = 1 in the case of relapse or death) or censoring information (*δ_i_* = 0), and the p-dimensional vector **x**_*i*_ defines the observed covariates of subject *i*. A subject is at risk if it undergoes an event or is censored. With *t*_1_ *< … < t_m_* being the ordered unique event times (with *δ_i_* = 1), at time *t_j_*, all at-risk individuals constitute the risk set *R*(*t_j_*), which is defined as the set of all observations with longer observation time *T_i_ > t_j_*.

In order to relate survival and observed covariates in our algorithm, we use the Cox proportional hazards model (Cox, 1972). In this model, the hazard for subject *i* is defined in semi-parametric form:

\begin{align*}h_i ( t \mid { \bf x_i} ) = h_0 ( t ) \exp \left(
\sum_{k = 1}^{p}{x_{i , k} \beta_k} \right) \tag{2.1}\end{align*}

where *h*_0_ is a common baseline hazard and ***β*** is a vector of regression coefficients of length *p*. Inference on ***β*** is performed by maximizing the partial likelihood, defined as

\begin{align*}L ( { \bf {\beta }} ) = \prod_ { i = 1 } ^ { m } {
\frac { \exp ( \sum_ { k = 1 } ^ { p } { x_ { i , k } \beta_k } )
}  { \sum_ { j \in R_i } { \exp ( \sum_ { k = 1 } ^ { p } { x_ { j
, k } \beta_{k} } ) } } } \tag { 2.2 } \end{align*}

where the baseline hazard *h*_0_(*t*) cancels out. The estimated risk score per subject is summarized by $\hat{ \eta_i} = \mathop\sum\nolimits_{k = 1}^p x_{k , i}  \hat{\, \beta_k}$, which expresses the linear combination of covariates with an estimated vector of coefficients $\hat{  \bf {\beta}}$.

Investigation of the prediction accuracy and feature selection in our framework is performed with the C-index definition (Uno et al., 2011), denoted as *C_Uno_*. The C-index of Uno for a prespecified point in time *τ* is defined as follows:

\begin{align*}C_ { Uno , \tau } = \frac { \sum_ { j , k } { \hat { G } ( T_j ) ^ { - 2 } I ( T_j < T_k ) I ( \hat { \eta_j } < \hat { \eta_k } ) \delta_j } }  { \sum_ { j , k } { \hat { G } ( T_j ) ^ { - 2 } I ( T_j < T_k ) \delta_j } } \in [ 0 , 1 ] \tag { 2.3 } \end{align*}

where *I*() is an indicator function. Here, $\hat{G} ( T_{j} )$ is estimated from the training data and is defined as the Kaplan–Meier estimator of the unconditional survival function:

\begin{align*}\hat { G } ( t ) = \prod_ { t_j \leq t } 1 - \frac { d_j }  { R ( t_j ) } \tag { 2.4 } \end{align*}

with *d_i_* being the number of events at *t_j_*. The *C_Uno_* index is estimated nonparametrically, thereby adjusting for the censoring bias via inverse probability weighting. A risk score *η_i_* is estimated for the selected features with new data **x**_*test*_ for each individual in the test set. This score is used as input for the *C_Uno_* function. To obtain the variation in *C_Uno_* with an independent test set, we calculated prediction performance with different random subsamples (of 90%).

An advantage of the *C_Uno_* approach compared to other C-index definitions (Heagerty and Zheng, 2005; Antolini et al., 2005) lies in its independence of the Cox proportional hazard assumption. The C-index can be interpreted as the probability of concordance between the predicted and observed survival times over all pairs of observations at a given time point. Similar to the standard binary AUC, a value of 0.5 indicates that the marker is not better than random guessing and a value of 1 represents perfect separation. In contrast to the standard area under the ROC curve, models with C-index of relatively low values (between 0.6 and 0.7) are often considered as having satisfactory predictive power. For example, a C-index of 0.67 was achieved (Tice et al., 2005) in a model predicting breast cancer based on genetic information, known as the Gail model (Gail et al., 1989). In cancer research, the absolute discrimination power is often not required; however, separation and classification of patients into groups of high and low risk is the primary goal. Therefore, this C-index is a favorable choice.

### 2.1. SurvRank

A schematic overview of the algorithm is shown in Figure 1, and further details are given in Algorithm 1. To fit a survival model and estimate generalizability, a repeated nested CV approach is used to first estimate the best number of features within an inner CV loop and then to estimate the performance of the model containing these features in an outer CV loop. Note that the identification of important features within the CV is based on different ranking methods.

#### 2.1.1. Feature ranking methods

Three approaches to generate ranked output lists of features were considered, that is, an approach based on the log-rank statistic (survCox), a Lasso-based approach for survival data (survLasso), and a randomized Cox model (survRand).

***Cox score ranking - survCox*** The Cox-based ranking approach sorts covariates according to their association with the survival response based on the Wald score test. For each feature, a univariate Cox model is fitted, and the obtained log-rank statistic is used as the ranking criterion (Moeschberger and Klein, 2003). A high test statistic indicates stronger association with the outcome. Note that this Cox score ranking is univariate in contrast to the other two approaches.

*L*_1_ ***norm (Lasso) ranking - survLasso*** In this approach, ranking is generated using a penalized *L*_1_ Cox regression (Tibshirani and others, 1997). Briefly, the *L*_1_ penalty (Lasso) in the Cox regression case seeks to find a solution for the following:

\begin{align*}\hat { \bold \beta } = \arg \min_ { \bold \beta } \left( \frac { 2 }  { n } ( \sum_ { i = 1 } ^ { m } { \bf x } _ { j ( i ) } ^T { \bold \beta } - \log \bigg( \sum_ { j \in R_i } { \exp ( { \bf x } _j^T { \bold \beta ) } \bigg ) } - \lambda \sum_ { i = 1 } ^ { p } { \mid \beta_i \mid } \right) . \tag { 2.5 } \end{align*}

An efficient implementation of the regularization path has been demonstrated (Simon et al., 2011). The complextity parameter *λ* determines the amount of shrinkage. The ranks of features are then obtained according to their appearance in the regularization path. All covariates not selected in the model obtain a rank that corresponds to the number of features *p*.

***Randomized Cox ranking - survRand*** This ranking method consists of a two-step procedure. In the first step, *L*_1_ penalization is used to preselect a smaller number of features (*p*_pre_ *< p*). The cut-off criterion in the Lasso is defined such that at least 95% of the deviance is explained at the end of the lambda sequence. In the second step, a sub-sampling approach (500 times) randomly chooses without replacement a smaller number of features and estimates a multivariate Cox model. To avoid convergence issues in the fitting procedure of the multivariate Cox model, the number of features in each subsampling step *n_sub_* is limited to the number of observations (*n_sub_* = *n/*3). Each feature in one subsampled Cox model yields a Z-statistic. The number of selections per feature is controlled by *p_pre_*, thereby leading to *p_pre_/*3 number of Z-statistics for each feature on average. Finally, by calculating the mean over all Z-score subsamples, a final feature score is derived and used for ranking in survRand.

#### 2.1.2. Nested CV for estimating generalizability—TASK 1

The full dataset *D* : = *D_i_* with *D_i_* : = (*T_i_*, *δ_i_*, *x_i_*) is split into training set $D^{ - cv_{out}}$ and test set $D^{cv_{out}}$ with index set *cv*_*out*_ and its complement.

**Inner CV** Inner CV is applied to only the training set $D^{ - cv_{out}}$ by performing a second CV stratification, thereby yielding inner training $D^{ ( - cv_{out} , - cv_{in} ) }$ and inner test set $D^{ ( - cv_{out} , cv_{in} ) }$. Then, one of the described ranking functions is applied to the inner training set. By adding one feature at a time (following the ranking), a Cox model is estimated using the inner training data and evaluated with *C_Uno_* for the inner test data. This procedure is performed until a predefined maximum number of features is achieved and is repeated for all inner CV folds. The best number of features is determined by averaging over all inner CVs and selecting the number of features that corresponds to the maximum mean *C_Uno_.*

**Outer CV** For the outer CV, one feature ranking is performed for the whole training set. Then, using the best number of features derived in the inner CV, a Cox model is estimated using the training set, thereby yielding effect estimates for the selected features. Using these estimates, the unbiased prediction performance with the unseen test set is quantified by *C_Uno_*, corresponding to TASK 1. Note that the entire procedure (including the inner CV) is applied to all outer CV folds.

**Repeated CV** To obtain an estimate of the variance of prediction accuracy, this approach is repeated *t*_*times* for different splits of the dataset.

#### 2.1.3. Final model—TASK 2

The repeated nested CV combined with stepwise feature selection based on the ranking function yields a ranked set of features for each CV run. In addition, the performance on the test set for each run is recorded (number of runs *K* = *cv_out_* × *t*_*times*). As these ranked lists of selected features are not necessarily the same, it is not clear how to aggregate them to a final set of features that can be used for predicting risk scores for new patients. Here, we propose an approach that leverages the information from all individual CV runs to determine a final set of features for which a final model can be fit.

Our weighted approach uses information from the outer CV performance corresponding to each run, thereby addressing TASK 2. The weight of run *i* is defined as follows:

\begin{align*}w_i = \begin{cases} \frac { 1 }  { K } \exp ( \log ( 2 ) \frac { devAUC_i }  { 0.1 } ) , \quad { \rm if } \ C_ { Uno , i } \geq 0.5 \\ 0 , \qquad\qquad\qquad\qquad\quad \, { \rm if } \ C_ { Uno , i } < 0.5 \end{cases} . \tag { 2.6 } \end{align*}

Here, *devAUC_i_* denotes the relative *C_Uno_* of an individual CV run compared to the average performance of all runs. The weights $w_i^{ \prime}$ are further normalized to sum to one ($w_{i}^{ \prime} = w_{i} / \Sigma w_{i}$). The final set of predictors is determined by majority voting as follows:

\begin{align*}I ( p_{j} ) = \begin{cases}1 \qquad \,\; { \rm if}
\ p_j  >  0.5 \\ \qquad \qquad \qquad \quad { \rm with} \ p_j =
\sum \limits_{i = 1}^{K}I ( j , i ) w_i^{ \prime} \\ 0 , \qquad {
\rm if} \ p_j \leq 0.5\end{cases} \tag{2.7}\end{align*}

where *I*(*j*, *i*) is 1 if the feature *p_j_* was selected in run *i*.

**Algorithm 1:** SurvRank algorithm with repeated nested CV

Finally, one survival model can be calculated with the selected features using the entire dataset, thereby leading to effect estimates $\hat{  \bold \beta}_{train}$ and risk scores for each subject. This is used to predict survival probabilities with unseen data with similar predictive power as estimated in the nested CV.

### 2.2. Comparison method

To compare this approach to existing methods, a commonly used regularized survival model based on Cox-Lasso was selected (coxLasso). For coxLasso, the same unbiased approach was performed to estimate the prediction accuracy with CV by applying the same repeated CV parameters. One CV step consists of separation into different folds and optimizing the penalization parameter $\hat{ \lambda}$ by the inner CV of one fold. This optimized $\hat{ \lambda}$ was used to predict the unseen test fold, thereby measuring performance with *C_Uno_*. For coxLasso, the final selection of covariates, which are used for prediction with the test set, was estimated by applying CV to the entire training dataset once. By optimizing the partial likelihood in the Cox regression, the number of features was obtained with cross-validated minimum deviance for coxLasso.

## 3. Results

### 3.1. Simulation and validation setup

To evaluate our algorithm, we generated a high-dimensional, multivariate normally distributed dataset with *n* = 100 observations and *p* = 500 features. The survival times *T_i_* followed an exponential distribution with mean $\eta_i = 1 / ( \lambda_T \mathop\sum\nolimits_{i_1}^{4} x_i \beta_i )$, which we set to *λ_t_* = 0.5 and *β*_1_ = 1.5, *β*_2_ = −1.5, *β*_3_ = −1, and *β*_4_ = 1 for our framework. An independent random censoring time *T_cens_* was simulated such that it followed an exponential distribution, which we fixed to mean 2. The observed survival times *T_obs_* are expressed by *T_obs_* = *min*(*T_cens_*, *T_i_*), which leads to independent censoring of approximately 50%. The maximum number of features was set to 30. Partitioning into training and test sets was applied in all configurations with the same parameters (*cv_in_* = 10, *cv_out_* = 10, *t*_*times* = 10). To calculate *C_Uno_*, we fixed *τ* at the last observed survival time.

We first used the simulated data to estimate generalizability accurately, which is directly related to TASK 1. By applying a final model fit on the training set and estimating the performance with 10 simulated test sets, we retrieved the performance of our model selection with new unseen data. Ideally, the performance difference between the training and test data should be small. Otherwise, we would have a classical overfitting situation with the training data, where generalization accuracy to new unseen test data is not fulfilled. This procedure was repeated for 100 different training datasets. Furthermore, we calculated the true *C_Uno_* for the training set and the test sets using only the true effects *β*_1_,…,*β*_4_.

We then attempted to retrieve the correct set of features, thereby addressing TASK 2 (feature selection). To achieve this, we calculated the *F*_1_ score, which is defined as the harmonic mean of precision and recall, that is, $F_{1} = 2 \times  \frac {{ { \rm precision } \cdot { \rm recall }} { { \rm precision } + { \rm recall } }} .$ Here, the *F*_1_ score was calculated to compare the selected features with respect to the four true features.

We then compared our approach with a commonly used regularized survival model. Here, we estimated a penalized survival Cox model with Lasso (coxLasso based on the R package glmnet).

### 3.2. Simulated dataset results

**FIG. 2.**
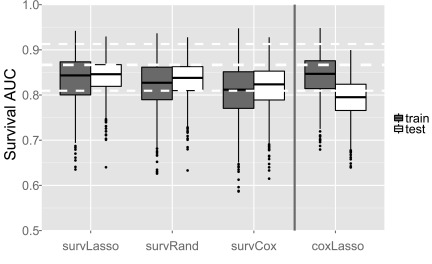
Prediction performance with simulated data. A total of 100 training datasets were simulated, and unbiased *C_Uno_*s were obtained for each repetition of the nested CV. For each of the 100 training datasets, 10 test sets were created to test prediction performance with new data. White dashed lines indicate the average of the true *C_Uno_* with the simulated datasets with an empirical 95% quantile range.

We observed good performance with the test data and comparable results for accuracy with the training set compared to the test sets (Fig. 2), thereby addressing TASK 1. The coxLasso approach performed similarly with the training data compared to survLasso from our package; however, as expected, prediction with unseen new data shows substantial overfitting. The survRand ranking function demonstrated higher variance of *C_Uno_* with the training set. survCox ranking performed worse with the training data; however, the final feature selection results showed comparable prediction accuracy with new test data. The overall worse performance of survCox illustrates the advantage of the multivariate ranking function of survLasso and survRand compared to survCox with univariate ranking.

**FIG. 3.**
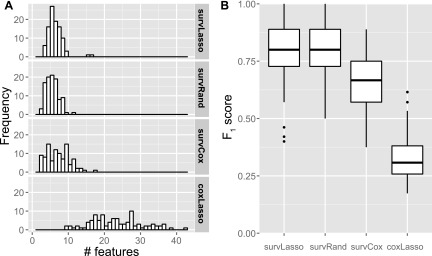
**(A)** Number of selected features across simulated training datasets in the weighted approach. **(B)** *F*_1_ scores for selected features to compare the selected features with the set of four true features.

Compared to standard coxLasso, the sparser set of selected features represents an advantage of our ranking and final feature selection approach (Fig. 3A), thereby addressing TASK 2. This illustrates that selecting features according to the data fit (deviance), as used in the standard coxLasso approach, produces too many selected features. In addition, we investigated whether the correct covariates were selected. We observed higher *F*_1_ scores (Fig. 3B) with our approach compared to coxLasso. These results illustrate the overfitting of the coxLasso approach, that is, it selects several random, noninformative features (resulting in a high FPR) and considerably overestimates predictive power with training sets (reduction of *C_Uno_* on average of 0.05 or 6% from training to test).

#### 3.2.1. Runtime evaluation

An important aspect for nested CV approaches is the required computation time. The SurvRank package inherently supports parallelization across multiple cores on the same machine. Table 1 shows the runtimes for different variable settings for the three ranking functions using a single core of an Intel Core i5 2.6 GHz CPU. Here, we observed that the number of features *p* scaled approximately linearly with computation time for survLasso, survRand, and coxLasso. survLasso was slower than coxLasso in the first two settings by a factor of approximately 2.5, taking the additional stepwise selection into account. Doubling the number of observations *n* increased computation time by a factor of 2.2 for survLasso and survRand and by a factor of 3.6 for coxLasso. In contrast, the computation time of survCox scaled approximately linearly with the number of features due to the univariate ranking procedure. For survCox, an increasing sample size increased computation time only slightly.

**Table 1.** Computation Time in Minutes for Different *p* × *n* Setups

p	100	200	200	500
n	100	100	200	100
survLasso	9.00	9.43	20.33	19.00
survCox	10.03	16.43	16.48	27.58
survRand	77.07	82.70	186.57	86.28
coxLasso	3.70	3.98	14.38	5.42

Parameters set to *t*_*times* = 10*, cv_out_* = 10, and *cv_in_* = 10.

### 3.3. Application to three breast cancer gene expression datasets

To evaluate our approach with real clinical data, we applied the pipeline to microarray datasets from breast cancer patients with survival information (relapse time) after surgery (mastectomy) or radiotherapy. We used two independent datasets to estimate the prediction accuracy with unseen data to assess how well our method performs with TASK 1. To identify a predictive subset of features, we used our approach with different ranking functions, thereby addressing TASK 2. In addition, we compared the performance of our approach to a standard CoxLasso model and a set of 76 marker genes identified in the primary publication (referred to as geneMarker). This geneMarker was derived by ranking the features according to an averaged Cox score (using bootstrap samples).

The first dataset contained 286 patients with lymph node-negative breast cancer. For each patient, information about estrogen receptor status positive (ER+) and estrogen receptor status negative (ER-) was recorded, assuming that disease progression differs for these subgroups. This first dataset served as the training set [accession number GSE2034 (Wang et al., 2005)]. Wang et al. identified a predictive set of 76 genes (geneMarker) composed of 60 genes for the ER+ group and 16 genes for the ER- group. We attempted to obtain an alternative sparse set of genes with better generalizability to evaluate the performance of our approach with two independent validation sets, that is, accession numbers GSE7390 (Desmedt et al., 2007) and GSE1456 (Pawitan et al., 2005). There was an overlap of 18,842 features across the three datasets. In the training data, there were 209 patient samples in the ER+ group and 77 observations with ER- status. The first test dataset (test set 1) consisted of 134 samples in the ER+ group and 64 in the ER- group. The second test set (test set 2) contained 125 subjects in the ER+ group and 27 in the ER- group. Due to the larger number of observations, we focused on the ER+ subgroup for our evaluation.

We applied our different ranking algorithms to the dedicated training set and obtained a final marker. Furthermore, the selected genes were evaluated with the new and unseen test sets. The parameters of the repeated nested CV were determined as *t*_*times* = 20, *cv_out_* = 10, and *cv_in_* = 10. The maximum number of features was set to 75, and *τ* in *C_Uno_* was set to 10 years.

The geneMarker and the coxLasso approach served as comparison models for our ranking algorithms. The results of geneMarker were calculated by applying ridge regression to the training data and then evaluating performance with the two test sets. For coxLasso, we repeated the final feature selection ten times to determine the optimal penalization parameter, because coxLasso depends on the sampling of CV folds.

### 3.4. Breast cancer data results

**FIG. 4.**
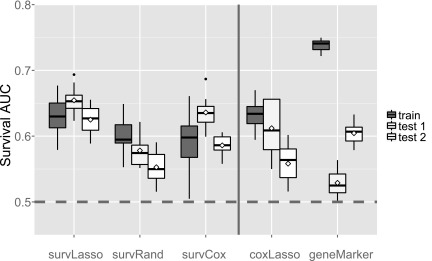
Prediction accuracy with three breast cancer data sets. The performance of the training data set was compared to two independent test sets for the ER+ group. Feature selection was based on the weighted approach. Diamonds show performance with the whole test set, whereas variation in the boxplots was obtained by subsampling the test data sets.

**FIG. 5.**
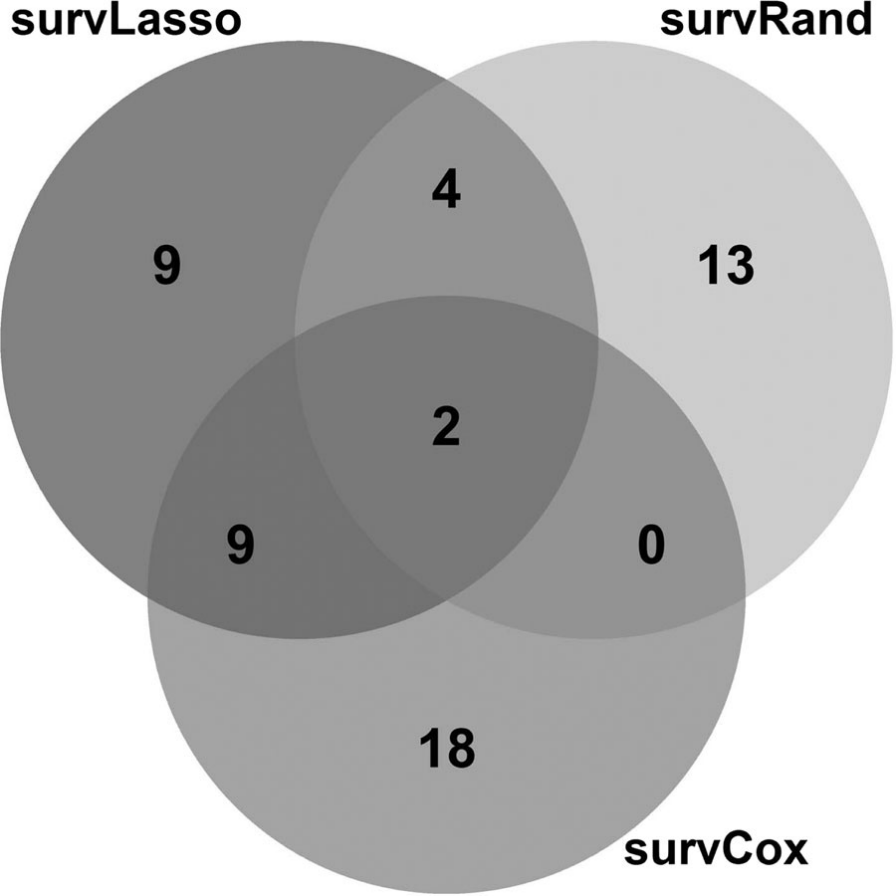
Overlap of selected genes of the different ranking functions.

For our approach, performance with the unseen test dataset showed similar prediction accuracy compared to the training data (Fig. 4). This indicates that our nested CV strategy was able to estimate the generalizability of the predictor correctly, thereby solving TASK 1. The number of selected features varied slightly between the three approaches of our package (24, 19, and 29 for survLasso, survRand, and survCox, respectively), thereby addressing TASK 2. survLasso and survCox showed larger overlap of selected genes compared to survRand (Fig. 5). As in the simulation study, survLasso performed considerably better than survCox (on average *C_Uno_* decreased by 0.03 or 5%), again illustrating the advantages of a multivariate ranking approach compared to univariate ranking. Similar to the results of the simulation study, coxLasso selected 53 features with too many false positives, resulting in a reduced performance with the test data sets. geneMarker resulted in clear overfitting of this marker set with the training dataset (as expected), where geneMarker was derived. Therefore, these results can be interpreted as training performance. Consequently, the predictive power decreased strongly with the test sets. Comparing the geneMarker set with the selected markers in survLasso, survRand, and survCox yielded a small overlap, that is, survLasso 2 genes, survRand 0, and survCox 5 (details in Supplementary Fig. S1, available online at www.liebertpub.com/cmb).

## 4. Discussion

We have proposed a new framework to reliably estimate prediction accuracy and generalizability and to select the most predictive features in a high-dimensional survival prediction setting. To avoid overfitting while selecting features with high predictive power, the proposed approach estimates accuracy and performs feature selection using repeated nested CV with novel feature combination heuristics.

Our approach differs from standard approaches, such as the CoxLasso approach, in two ways. First, the selection of features is determined by the best predictive feature combination (using *C_Uno_*) rather than the best data fitting combination, thereby reducing the risk of overfitting. Second, for final feature selection, our approach leverages information from different CV runs. The CoxLasso approach uses the minimum cross-validated deviance of the whole dataset, while the proposed approach aggregates the results of different CV runs and applies a weighting scheme to select only predictive features. This combination of aggregating CV runs by weighting results in sparser feature selection with more accurate estimation of predictive power.

Using simulated data, we demonstrated that the proposed method can identify true features and can correctly estimate prediction accuracy with new data without overfitting. By comparing the results of different methods in this simulation setup, we observed that survLasso dominates survCox with training and test data. This effect can be explained by the multivariable ranking procedure of survLasso (considering all features) in contrast to the univariate ranking of survCox, which treats features independently.

With breast cancer data, our pipeline based on two of our ranking approaches was able to estimate similar prediction performance with the test datasets compared to the training data. However, the survRand approach showed a drop in prediction performance with the breast cancer test data. This effect is illustrated in Figure 5, where we observe that this ranking approach has only small overlap compared to survLasso and survCox. The 19 selected features in this approach lead to lower prediction performance. By comparing coxLasso and survRand, we observed an overlap of six features that are only picked by these methods (Supplementary Fig. S1), thereby introducing noise to the model. In addition, the sampling strategy of survRand might introduce some noise to the selection process. This again confirms the robust performance of survLasso compared to the other ranking methods.

Our approach can be extended in several directions. (1) In clinical applications, variables such as age, gender, height, and BMI are collected routinely. Therefore, it would be desirable to force such features into the model and evaluate the additional benefit of omics data. (2) Our framework uses the Cox proportional hazards model. Extending the approach to accelerated failure time models or frailty models may improve the baseline hazard estimation, such as time-varying hazards or random effects. (3) Applying repeated nested CV to classification tasks may also be an interesting extension.

Importantly, our approach as a biomarker discovery method focuses on identifying a predictive biomarker combination and does not provide functional interpretation of the selected features (e.g., genes and transcripts). Therefore, we recommend using the SurvRank package with the survLasso approach and weighted final feature selection, due to the low computational demands and best results from both the simulation study and the clinical data.

In summary, we provide a flexible, ready-to-use toolbox for survival data that allows for unbiased estimation of prediction accuracy for survival models and extracts the most predictive features from high-dimensional survival datasets.

## Supplementary Material

Supplemental data
