# Supplementary figures and images for: Unbiased Prediction and Feature Selection in High-Dimensional Survival Regression
Source: J Comput Biol. 2016 Apr 1;23(4):279–90. doi: 10.1089/cmb.2015.0192 (PMC4827277; doi:10.1089/cmb.2015.0192)

## Supplementary Data

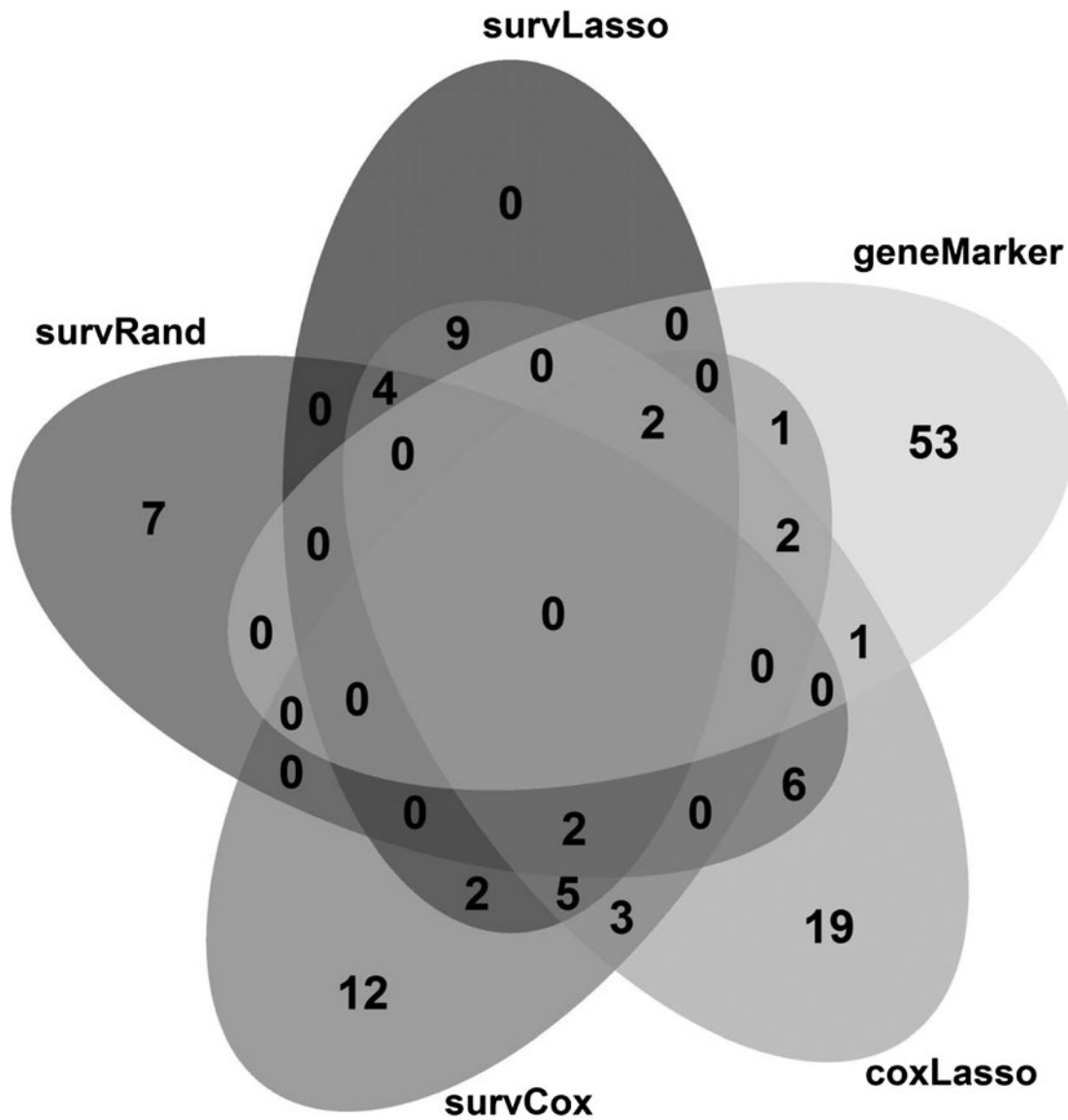

**SUPPLEMENTARY FIG. S1.** Overlap of the selected genes for all comparisons.

Supplement: Supplemental data [file Supp_Fig1.pdf]
